# Global scale phylogeography of functional traits and microdiversity in *Prochlorococcus*

**DOI:** 10.1038/s41396-023-01469-y

**Published:** 2023-07-15

**Authors:** Lucas J. Ustick, Alyse A. Larkin, Adam C. Martiny

**Affiliations:** 1 Department of Ecology and Evolutionary Biology, University of California Irvine, Irvine, CA 92697 USA; 2 Department of Earth System Science, University of California Irvine, Irvine, CA 92697 USA; 3 Present Address: Structural and Computational Biology Research Unit, European Molecular Biology Laboratory, Heidelberg, Germany; 4 Present Address: Global Ocean Monitoring and Observing, National Oceanic and Atmospheric Administration, Washington, DC USA

**Keywords:** Microbial ecology, Water microbiology

## Abstract

*Prochlorococcus* is the most numerically abundant photosynthetic organism in the surface ocean. The *Prochlorococcus* high-light and warm-water adapted ecotype (HLII) is comprised of extensive microdiversity, but specific functional differences between microdiverse sub-clades remain elusive. Here we characterized both functional and phylogenetic diversity within the HLII ecotype using Bio-GO-SHIP metagenomes. We found widespread variation in gene frequency connected to local environmental conditions. Metagenome-assembled marker genes and genomes revealed a globally distributed novel HLII haplotype defined by adaptation to chronically low P conditions (HLII-P). Environmental correlation analysis revealed different factors were driving gene abundances verses phylogenetic differences. An analysis of cultured HLII genomes and metagenome-assembled genomes revealed a subclade within HLII, which corresponded to the novel HLII-P haplotype. This work represents the first global assessment of the HLII ecotype’s phylogeography and corresponding functional differences. These findings together expand our understanding of how microdiversity structures functional differences and reveals the importance of nutrients as drivers of microdiversity in *Prochlorococcus*.

## Introduction

Microbial communities harbor vast phylogenetic diversity that is tightly linked to biogeographic partitioning of biomes. High resolution microbial diversity has been revealed through advances in sequencing technologies [1–5]. Fine scale phylogenetic differences, termed microdiversity (greater than 97% 16S rRNA gene similarity), have been shown to distinguish physiologically unique populations [6, 7]. Moreover, these diverse microbial populations harbor distinct functional traits conserved across various phylogenetic depths [8]. Microdiversity partitions important microbial traits such as antibiotic resistance, toxin production, nutrient uptake, and phage resistance [9–12]. While there is a clear parallel between fine phylogenetic clusters and differential genome content [13], less is known about the specific functional differences associated with these lineages. Functional differences between closely related microbes can confer significant competitive advantages and niche specialization [12]. Thus, to better understand the eco-evolutionary processes that drive differentiation between closely related microbial populations, it is critical that we link phylogenetic microdiversity with specific functional adaptations.

In the numerically dominant phytoplankton *Prochlorococcus*, we observe a clear phylogenetic organization of traits [14, 15]. Early in the evolutionary history of *Prochlorococcus*, a high light (HL) phylogenetic group splits from a diverse set of low light (LL) adapted clades [6]. Within the high light clade, *Prochlorococcus* demonstrates further partitioning with a split between a high (HLII) and low temperature ecotype (HLI) [16, 17]. Within these well-established ecotypes, we observe phylogenetic microdiversity that follows clear spatial differentiation [18–20]. This microdiversity has been linked to differences in genome content, but the specific functional differences are unknown [21]. Niche differences between microdiverse sub-clades have largely been explained by correlative environmental relationships [22, 23]. It has been hypothesized that differences in adaptation to nutrient limitation (specifically to phosphorus and nitrogen limitation) may be associated with different lineages, but no clear connection between fine-scale phylogenetic differences and functional gene content have been identified in *Prochlorococcus* [24–26].

The *Prochlorococcus* ecotype HLII is globally distributed and highly abundant in warm oligotrophic regions, making it a good model for studying the organization of microdiversity. Adaptations to low nutrient conditions vary spatially and are indicative of local nutrient conditions [27], but the presence/absence of these genes lack phylogenetic organization in our current ecotype groupings [24–26]. While specifically exploring the phylogenetic distribution of *narB* (nitrate reductase) and *nirA* (nitrite reductase), the presence/absence of these genes did not display a clear evolutionary structure and had a sporadic distribution [24]. It was suggested that this structure arose through vertical evolution of the traits from basal lineages followed by gene loss events. An alternate hypothesis is that nutrient acquisition traits are shared through horizontal gene transfer and thus do not follow a clear phylogenetic pattern [25, 28, 29]. It is thus unclear whether nutrient adaptation is phylogenetically conserved at the microdiverse phylogenetic level, and how adaptation to variable nutrient conditions influences the biogeographic distribution of these populations.

**Fig. 1:**
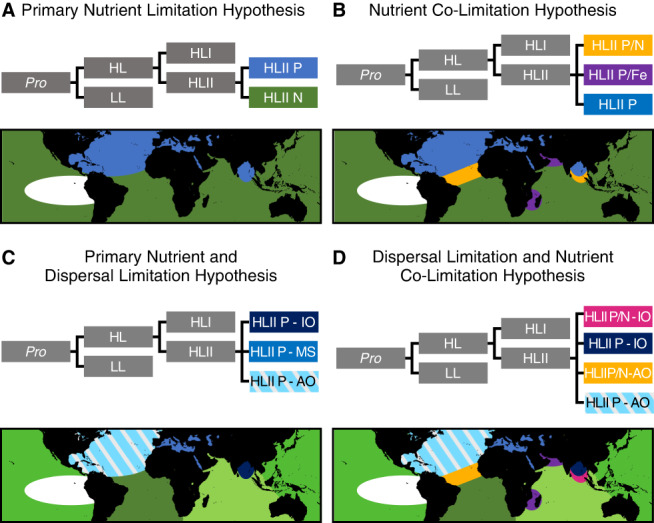
Hypotheses on the drivers of microdiversity and predicted global distributions. Hypotheses vary based on two different factors: nutrient conditions (single nutrient limitation vs. co-limitation) and dispersal limitation (dispersal limited vs. effectively globally distributed). Colors on the trees and maps correspond across all panels and represent the expected phylogenetic structure and distributions based on each hypothesis. **A** Primary nutrient limitation hypothesis. **B** Nutrient co-limitation hypothesis. **C** Primary nutrient and dispersal limitation hypothesis. **D** Dispersal limitation and nutrient co-limitation hypothesis. Abbreviations represent hypothetical haplotypes based on both limiting nutrient type and general region: Phosphorus (P), Nitrogen (N), Iron (Fe), the Indian Ocean (IO), the Mediterranean Sea (MS), and the Atlantic Ocean (AO). For example HLII P-AO is a hypothetical low P adapted haplotype found only in the Atlantic Ocean.

Here, we aim to link the global microdiversity and phylogeography of *Prochlorococcus* with population-specific functional diversity. Specifically, we isolated reads that mapped to a well-studied phylogenetic marker gene to capture the phylogeography of *Prochlorococcus* and simultaneously quantified differences in the functional *Prochlorococcus* gene content from surface ocean metagenomes. We then generated metagenome-assembled genomes (MAG) of the novel haplotypes identified. We hypothesize that functional differences within *Prochlorococcus* HLII is primarily driven by adaptation to different nutrient regimes (Fig. 1). We present a hierarchical set of hypotheses regarding the phylogenetic structure of *Prochlorococcus* HLII microdiversity. The hypotheses are based on two different factors: how do nutrient conditions (primary nutrient limitation type vs. co-limitation) and dispersal limitation (dispersal limited vs. effectively globally distributed) drive microdiversity. The first hypothesis predicts microdiversity is driven by differences in adaptation to the primary limiting nutrient (Fig. 1A). In this case, we expect globally dispersed haplotypes that are differentiated based on single nutrient conditions, i.e., nitrogen (N) limitation vs. phosphorus (P) limitation. Our second hypothesis predicts microdiversity is driven by combinatorial differences in nutrient limitation type (Fig. 1B). Like the previous hypothesis, we expect unique haplotypes for each type (N, P) of primary nutrient limitation but also unique haplotypes for different combinations of co-limitation such as N/P limitation vs. N/Fe limitation. The third hypothesis predicts microdiversity is driven by the primary limiting nutrient and dispersal-limited genetic drift (Fig. 1C). In this case, proximity of samples and the primary limiting nutrient are the strongest drivers of microdiversity. For example, we expect P limited populations in the North Atlantic Ocean to have phylogenetically distinct core genes from P limited populations in other oceans. The final hypothesis predicts microdiversity is driven by both limiting nutrient type, nutrient co-limitation, and will be dispersal-limited (Fig. 1D). In this case, we expect regionally bounded populations, but both the primary limiting nutrient and co-limiting nutrients lead to distinct populations.

## Methods

### Metagenomes

We analyzed surface metagenomes (<25 m depth) from Bio-GO-SHIP [30], and GEOTRACES [31].

### Read recruitment and quality filtering

Raw metagenomic reads were quality filtered and adapter sequences were removed using Trimmomatic v0.35 [32]. Metagenomic reads were recruited using Bowtie2 v2.2.7 [33]. The reads were recruited to a reference database comprised of 115 genomes with representatives of each major ecotype of *Prochlorococcus* and as well as *Synechococcus*, *Pelagibacter* and *Roseobacter* to help reduce mis-recruitment of closely related microbes (Table S3). Bowtie2 was used with the following flags --no-unal --local -D 15 -R 2 -L 15 -N 1 --gbar 1 --mp 3. Resulting SAM files were sorted and indexed with samtools v1.3 into BAM files [34].

### Profiling recruited reads

Recruited reads were profiled using Anvi’o v5 [35]. All open reading frames in the reference database were aligned and clustered using NCBI BLAST [36] and MCL [37] following the Anvi’o Pangenomic workflow [38]. All gene clusters from the HLII genomes were extracted and separated into single copy core genes (SCCG) and genes in the flexible genome (non-SCCG).

### Metagenomic rpoC1 consensus sequences

Reads which recruited to the *rpoC1* gene across all reference genomes were extracted. Reads were then separated by sample and ecotype and all reads that mapped to HLII reference genomes were aligned to a reference *rpoC1* sequence. Based on the alignment we calculated a consensus *rpoC1* sequence for the HLII ecotype for each sample in our dataset. Consensus sequences were made by aligning with Bowtie2 [33], then the consensus was calculated and quality controlled by samtools [34]. Only samples that passed the following quality metrics were used in all further analysis: a minimum of 2000 reads that mapped to *rpoC1*, minimum of 5x SCCG coverage of HLII, and the sample must have at least 90% of SCCG reads binned as HLII. Consensus sequences and metadata can be found in Data S1.

### Gene analysis

Sequence coverage for all non-SCCG’s were normalized by average SCCG coverage. This roughly estimates the number of copies of a gene per individual genome in the sample. We calculated a *z*-score for the normalized abundance of each gene and the resulting normalized coverage was analyzed using principal component analysis (PCA) in R [39]. Then we collected the top 100 genes that contributed to the first four principal components and extracted the NCBI COG annotations for each gene from the Anvi’o profile summary files. In addition, we identified the nutrient acquisition and metabolism genes shown in Table S2 and extracted the loadings of these genes from the PCA analysis. Then the average magnitude and angle of each gene’s eigen vector was calculate based on groupings by nutrient type (P, N, Fe). This average vector was overlayed on the PCA along with the fit of in situ sea surface temperature measurements calculated using the envfit command from the vegan package in R.

### Metagenomic rpoC1 consensus analysis

Consensus sequences were analyzed using R [39]. Consensus sequences were transformed into binary sequences. Phylogenetic distances between samples were calculated using a binary Jaccard from the Vegan R package [40]. The sequences were hierarchically clustered using hclust with the McQuitty method. The number of stable clusters were selected by minimizing the sum of squares within each cluster. Resulting clustering was then bootstrapped 1000 times and clusters that were in less than 60% of the bootstraps were removed from further analysis.

### Nutrient stress indicator

We used a genomic indicator of nutrient stress termed (Ω) derived from *Prochlorococcus* populations. Macronutrients are below detection limits in much of the oligotrophic surface ocean making this indicator especially useful in these regions. The indicators have been linked to surface nutrient concentrations and have been previously described [27, 41, 42]. We followed the same pipeline described in [27] and used alkaline phosphatase genes (*phoA, phoX*) for P-stress (Ω P), nitrite and nitrate assimilation and uptake genes *(focA, moaA-E, moeA, napA, narB, nirA*) for N-stress (Ω N), and Fe uptake transporter genes (*cirA, expD, febB, fepB/C, tolQ, tonB*) for Fe-stress (Ω Fe). The coverage of each gene was normalized to *Prochlorococcus* single copy core gene coverage (SCCG). We then calculated a *Z*-score for each gene and took the average across each nutrient type (P, N, Fe).

### Amplicon sequence analysis

The relative abundance of amplicon derived *Prochlorococcus* haplotypes in the Indian Ocean were collected from [43] This study was also a part of the I09 Bio-GO-SHIP cruise and was collected at the same times and locations as the metagenomic samples. We compared the relative abundances of amplicon sequences grouped based on our metagenome consensus sequences *rpoC*1 and quantified the relationship using a *t*-test. This was calculated in R [39]. We also compared the genomic PCA values to the amplicon derived haplotype relative abundance. We quantified the relationship by calculating the Pearson correlation between the two measurements in R.

### Statistical analysis

Spearman correlation between genomic principal components and environmental factors, and *t*-tests between phylogenetic clusters were calculated in R [39]. Pairwise physical distance between samples was modeled in R. The distance was calculated by transforming a global map into a raster image with 400 rows and 800 columns (~0.45°). Raster squares that fell on land were made impassible and the shortest distance was calculated between all samples in a pairwise fashion. We then took our distance matrix and decomposed this into a single continuous component using metaMDS in R. This was done so the distance effect could be included in our PERMANOVA analysis. Environmental variables were correlated to phylogenetic distance and functional distance using PERMANOVA analysis with the adonis2 package in R. Distance decay was estimated by extracting the slope of a linear model between genomic/phylogenetic distance and physical distance. This was done in R with the lm function.

### Metagenome-assembled genomes

Metagenome-assembled genomes (MAGs) were created using the following pipeline. The reads were quality controlled using the same method described previously in the Read Recruitment and Quality Filtering section. Raw assemblies were made using the de novo assembler SPAdes with default parameters [44]. All 51 samples within the HLII-P cluster were assembled individually using the metaSPAdes assembly pipeline. Resulting assembled contigs were binned using MetaBAT2 with the default parameters [45]. MAGs were quality controlled and rapidly assessed using checkM [46]. We selected and annotated MAGs that were at least 60% complete with less than 0.3% contamination as reported by checkM (Table S4). MAG annotations were made by aligning the resulting bins against a reference of phosphorus, nitrogen, and iron acquisition genes using BLAST [36]. Gene annotations were used to cluster cultured genomes and the I09-1 MAG. Presence and absence of phosphorus, nitrogen, and iron acquisition genes were turned into binary values. We then calculated the pairwise Euclidean distance between samples, and then calculated the tree with hclust using the complete method in R.

### MAGs phylogenetic analysis

A phylogeny of the MAGs and cultured genomes was created using the *rpoC1* gene. Sequences of the *rpoC1* gene were extracted from all *Prochlorococcus* reference genomes and a single *Synechococcus* genome as an outgroup. The sequences were aligned using Mega7 [47] and Muscle [48]. The tree model was selected based on the maximum likelihood fit of 24 different nucleotide substitution models using MEGA7. GTR + G + I was selected since it had the lowest Bayesian Information Criterion and Akaike Information Criterion values. The phylogenetic tree was created using raxml [49] with the following arguments raxmlHPC -f a -x 318420 -p 318420 -N 100 -m GTRGAMMAX -O -o GEYO-Syn -n out_file -s align_file -w out_dir. GEYO was a *Synechococcus* genome used as an outgroup. The resulting tree was visualized using iTOL [50].

## Results

To link phylogenetic and functional diversity at the microdiverse population level, we analyzed 630 surface ocean metagenomes from Bio-GO-SHIP [30] and GEOTRACES [31] and isolated reads that mapped to *Prochlorococcus* HLII reference genomes (Fig. S1). We first characterized the global functional diversity within HLII by quantifying the variation in gene frequency across the global ocean, which we will refer to as genomic diversity in this study. We then related this genomic diversity to phylogenetic changes by identifying dominant single nucleotide polymorphisms (SNPs) among *Prochlorococcus* populations, which we refer to as phylogenetic diversity. Based on the relationship of genomic and phylogenetic diversity, we then created targeted metagenome-assembled genomes to better understand the genomic structure of each distinct SNP derived phylogenetic cluster.

**Fig. 2:**
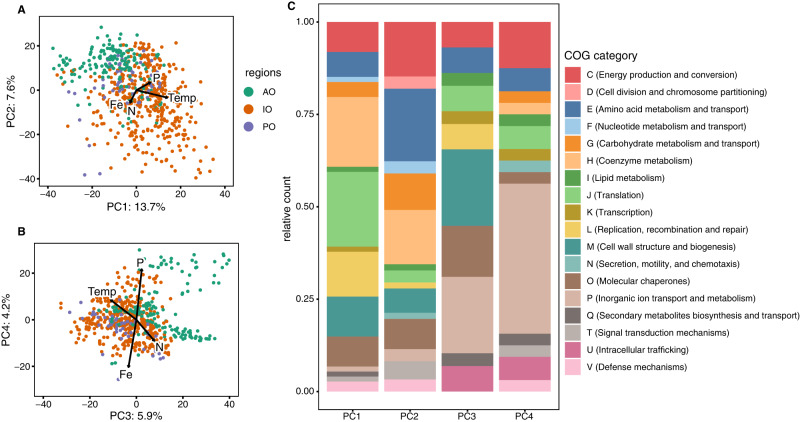
Global variation in gene content (genomic diversity) of *Prochlorococcus* HLII. **A**, **B** Principal component analysis of HLII gene abundance (genomic diversity). Vector length of sea surface temperature is based on variance explained by the environmental factor, while Fe, P, and N is the average vector of nutrient acquisition genes grouped by type. Samples are colored by ocean basin Atlantic Ocean (AO), Indian Ocean (IO), and the Pacific Ocean (PO). **C** 100 most informative genes for each principal component based on PCA loadings grouped by cluster of orthologous groups (COG) annotations.

*Prochlorococcus* genomic diversity revealed clear separation between major ocean regions. We performed a PCA analysis on the normalized abundance of the variable gene content within the HLII ecotype. To contextualize this dimensional reduction, we fit the PCA with in situ sea surface temperature and overlayed the average direction and magnitude of nutrient acquisition and metabolism genes grouped by type (Fe/P/N) (Fig. 2A, B). The top 100 genes that contributed to each principal component were identified and counted based on NCBI clusters of orthologous groups annotations (COGs). PC1 captured 13% of the total variance in gene content and had the strongest correlation with temperature (Spearman *ρ* = 0.74, *p* < 0.05) (Figs. 2A and S2A and Table S1). PC1 was primarily informed by genes annotated as H (coenzyme metabolism), J (translation), and L (replication recombination and repair) (Fig. 2C). Three of the top five genes ordered by PC1 loadings were tRNA synthetases (Cysteinyl-tRNA synthetase, Histidyl-tRNA synthetase, and Seryl-tRNA synthetase). This along with a temperature correlation suggested these genes represented differences in energetic adaptations. PC2 captured 8% of the total variance and was enriched in the Atlantic but depleted in the Indian Ocean Samples (Figs. 2A and S2B and Table S1). PC2 was primarily informed by genes annotated as E (amino acid metabolism and transport), G (carbohydrate transport and metabolism), and H (coenzyme metabolism). PC3 captured 6% of the total variance and linked to genes annotated as M (cell wall/membrane/envelope biogenesis) (Figs. 2 and S2C). PC4 captured 4% of the total variance and was mainly informed by genes annotated as P (inorganic ion transport and metabolism). Of the top 50 genes ranked by PC4 loadings, 10 were involved in phosphorus acquisition and metabolism with positive loadings, and 4 were iron acquisition genes with negative loadings (Table S2). This relationship was further highlighted as the frequency of all acquisition and metabolism genes for phosphorus also had a strong positive relationship with PC4 (Spearman *ρ* = 0.88, *p* < 0.05) and the average abundance of all iron genes had a negative relationship (Spearman *ρ* = −0.79, *p* < 0.05) (Figs. 2B and S2D and Table S1). Overall, functional gene content was correlated with differences in temperature and nutrient regimes leading to distinct ocean basin distributions.

**Fig. 3:**
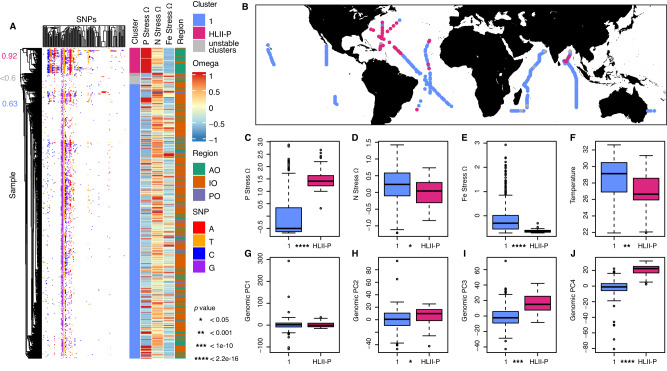
Global *rpoC1* derived phylogenetic diversity of *Prochlorococcus* HLII. **A** Clustering of the *rpoC1* marker gene based on sequence similarity, with corresponding region and metagenomically derived nutrient stress (Ω). Columns represent different single nucleotide polymorphisms (SNPs) with rows showing different metagenomic samples. Bootstrap values are color coded to match their corresponding cluster. **B** Spatial distribution of groups based on the clustering of *rpoC1* consensus sequences. **C**–**J** Comparison between phylogenetic clusters. **C**–**E** Differences in nutrient gene abundances. **F** Sea surface temperature between clusters. **G**–**J** Genomic PCA (distance based on differential gene abundance) differences between phylogenetic clusters. * Denotes *t*-test *p* value significance levels, *<0.05, **<0.001, ***<1e−10, ****<2.2e−16.

Phylogenetic microdiversity of *Prochlorococcus* populations also showed systematic biogeographic distributions. We identified 114 SNPs in the marker gene *rpoC1* globally (Fig. 3A). Based on the presence and absence of these SNPs, we detected two stable clusters with strong support (63 and 92% bootstraps) and two unstable clusters (51 and 10% of bootstraps) (Fig. 3A). The majority cluster was globally ubiquitous (cluster 1, defined in 63% of bootstraps). A second highly conserved cluster (defined in 92% bootstraps and had 14 unique SNPs) was detected primarily in the North Atlantic Ocean (lat: 16–39° N, lon: 52–71° W) and intermittently in the NE Indian Ocean (lat: 9–13° N lon: 85–89° E) (Fig. 3A, B). This cluster also appeared sporadically in the central and SW Atlantic Ocean. We compared the average relative abundance of the stable SNP clusters against nutrient stress genes grouped based on nutrient type (P, N, Fe), termed Ω (Fig. 3C–E) [27]. We labeled the second cluster HLII-P, because it was associated with significantly higher abundance of phosphorus genes than cluster 1 (ΩP mean HLII-P = 1.5, Cluster 1 = −0.5, *p* < 2.2e−16) as well as significantly lower abundance of iron genes (ΩFe mean HLII-P = −0.6, Cluster 1 = −0.03, *t*-test *p* < 2.2e−16) (Fig. 3A, C, E). Genomic and phylogenetic distances were significantly positively correlated although only 14% of genomic was redundant with phylogenetic distance (*R*^2^_mantel_ = 0.14, *p* < 0.001). Cluster HLII-P had a significantly higher functional genome PC4 values than cluster 1 (Cluster 1 PC4 mean = −1.60, HLII-P PC4 mean = 20.79, *t*-test *p* < 2.2e−16), suggesting that HLII-P spatially co-occurred wherever PC4 was enriched (Figs. 3A, B, J and S2). There were also significant differences in N genes (*t*-test *p* < 0.05), sea surface temperature (*t*-test *p* < 0.001), functional genome PC2 (*t*-test *p* < 0.05), and functional genome PC3 (*t*-test *p* < 1e−10), but all these factors were less significant than the aforementioned trends. Based on these observations, we hypothesized that the functional genome PC4 represented the phylogenetically conserved functional type of HLII-P. Thus, the phylogeography of our samples indicated a strong link between phylogeny and phosphorus limitation.

A comparison between our metagenomically derived haplotypes and amplicon sequences revealed consistency between the two assessments. We evaluated our metagenomically derived phylogenetic clusters against previously characterized amplicon derived haplotype abundances in the Indian Ocean [43]. These Indian Ocean haplotypes were derived using the relative abundance of mutually exclusive SNPs in amplicon sequences of the full *rpoC1* gene. This comparison was made to both validate the metagenomic results and to try and identify whether the HLII-P cluster was a result of one *Prochlorococcus* population or multiple co-existing populations. The HLII-P cluster was closely associated with the amplicon derived IO HLII.2 haplotype and had significantly higher relative abundances of the haplotype compared to samples in Cluster 1 (*t-*test, *p* = 1.547e−06) (Fig. S3). All samples in the HLII-P cluster had over 50% relative abundance of the amplicon derived IO HLII.2 haplotype. Differences in gene content (Genomic Diversity PC4) also had a significant linear correlation with the relative abundance of IO HLII.2 (Pearson *r* = 0.53, *p* < 0.001) (Fig. S3D). This together suggested the metagenomically derived HLII-P cluster was a result of a single *Prochlorococcus* population.

**Fig. 4:**
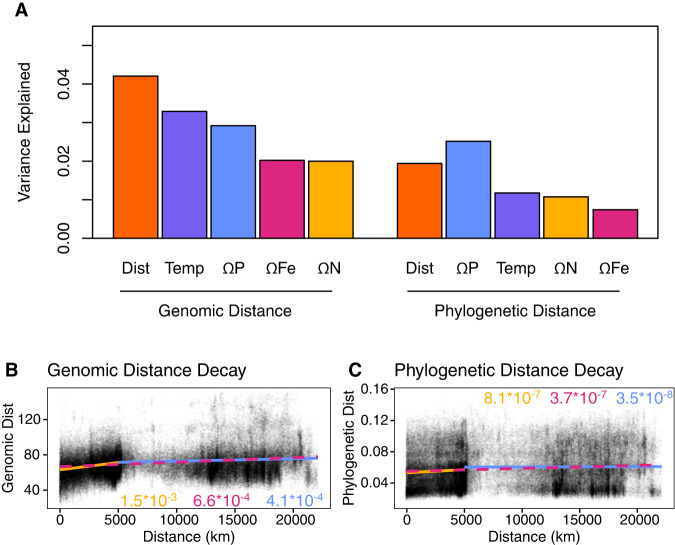
Variance explained by environmental factors and distance model. **A** Variance explained by environmental factors based on PERMANOVA. Variables are presented in the same order as they were used in the model. All relationships shown are significant (*p* value < 0.01). Distance, temperature, P-stress (ΩP), N-stress (ΩN), and Fe-stress (ΩFe) were included in the PERMANOVA. Distance decay of genomic distance (distance based on differential gene abundance) (**B**) and phylogenetic distance (distance based on *rpoC1* consensus sequences) (**C**). Sample points colored based on scatterplot density. Overall linear fit (red and dashed), within sampling transect linear fit (yellow), and between sampling transects linear fit (blue). Slopes of fits shown within figure.

Global shifts in functional and phylogenetic microdiversity within *Prochlorococcus* HLII were explained by unique environmental factors. We calculated the variance in functional diversity and phylogenetic diversity explained by a variety of independent variables (Fig. 4A). In our PERMANOVA analysis, we first captured the variance explained by physical distance to remove any distance effects. Then we ordered the remaining variables based on the variance explained, ordering from most to least variance explained. 4.2% of functional diversity and 1.9% of phylogenetic diversity could be explained by spatial distance between samples (Fig. 4A). Once distance effects were accounted for, temperature explained the most variance in genomic diversity (3.2%), while P gene abundance (Ω P) explained the most phylogenetic diversity (2.5%) (Fig. 4A). Overall, we could explain more of the genomic diversity (15%) with our environmental factors than phylogenetic diversity (8%). We also observed a stronger distance decay relationship between samples based on genomic distance (slope = 6.6*10^−4^) than phylogenetic distance (slope = 3.7*10^−7^) (Fig. 4B, C). Overall, we observed different factors correlated with genomic differences vs. phylogenetic differences, with genomic distance following regional differences such as temperature gradients, while phylogenetic variation was clearly linked to phosphorus limitation.

**Fig. 5:**
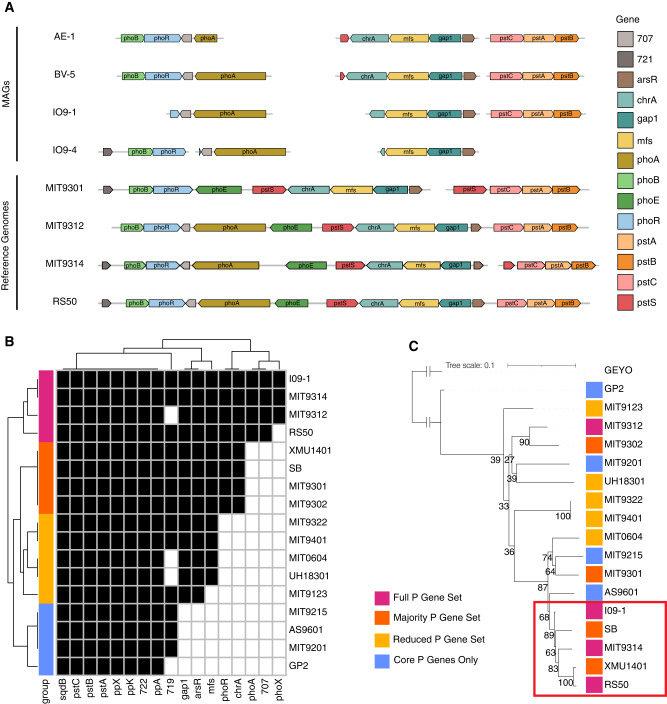
*Prochlorococcus* HLII-P MAG annotations of P acquisition genes and comparison to cultured genomes. **A** P acquisition gene presence and organization in MAG’s and cultured genomes. Annotated genes encode: putative arsenate stress transcriptional regulator (*arsR*), transporters (*chrA, mfs*), glyceraldehyde-3-phosphate dehydrogenase (*gap1*), alkaline phosphatase (*phoA*), P stress response (*phoB,E,R*), ABC-type phosphate transport system (*pstA,B,C,S*), upregulated under P stress (707,721). **B** Clustering of genomes based on presence/absence of P acquisition genes present in the MAG I09-1. **C** Phylogenetic tree based on the *rpoC1* gene with the *Synechococcus* genome GEYO as an outlier. Bootstraps shown at the corresponding node.

Metagenome-assembled genomes (MAG) supported that HLII-P represented a phylotype adapted to low phosphorus conditions. We assembled MAGs in all 51 samples within HLII-P to better understand the phylogenetic conservation of the P stress lineage of *Prochlorococcus*. Because of the relatively high diversity of *Prochlorococcus*, it is notoriously difficult to assemble, but we focused on assemblies that were at least 60% complete with less than 0.3% contamination and contained the P acquisitions genes. Our assembly resulted in 18 *Prochlorococcus* MAGs with 5 high-quality MAGs that passed our quality control (Table S4). Each high-quality MAG revealed a P acquisition gene ordering consistent with other HLII reference genomes (Fig. 5A). Additionally, four of the high-quality MAGs contained the *phoA* gene otherwise only found in *Prochlorococcus* HLII cultures isolated from chronically P limited regions (MIT9314, MIT9312, RS50). The parallel genomic structure of phosphorus genes in our MAGs and the reference genomes suggested a common genetic origin. We compared the presence/absence of the phosphorus acquisition genes found in MAG IO9-1 with cultured *Prochorococcus* HLII genomes (Fig. 5B). We observed a hierarchy of gene presence/absence and grouped the genomes based on this pattern (Fig. 5B). IO9-1 was the only MAG included in this analysis, because it was the only MAG with a fully assembled *rpoC1* marker gene. A phylogenetic analysis based on the *rpoC1* gene revealed a clade that contained genomes with the full or majority P acquisition gene set including MIT9314, RS50, and the IO9-1 MAG within HLII-P (Fig. 5C). The only exception was MIT9312, but this strain was isolated at 135 m depth. This position was likely due to the additional phylogenetic structuring between surface and deeper populations within HLII [51]. We performed the same analysis for N acquisition and Fe acquisition genes on the cultured genomes but did not find any organization within HLII (Fig. S4). Thus, our analysis of HLII-P MAGs revealed a sub-ecotype organization of P acquisition genes that corresponded to a clade within our cultured genomes.

## Discussion

We observed widespread functional genome diversity variation with a partial link to phylogeny. As previous studies observed a connection between microdiversity in *Prochlorococcus* and resource limitation, we hypothesized within-ecotype functional differences would mirror these patterns and primarily be driven by differences in nutrient conditions [15, 43]. In our analysis, temperature explained the most variation in genome content within the HLII ecotype, but adaptation to P limitation had the strongest link to phylogeny (Fig. 4). We hypothesized that differences in genomic diversity would be primarily explained by nutrient conditions. However, this hypothesis deserves revision given overall genome content was better explained by temperature (Figs. 1 and 4). This result was unexpected because temperature adaptation differentiates the HLII clade (high temperature) from the HLI clade (low temperature), but consistent with previous amplicon-based analyses that identified microdiverse clades within HLII further partitioing temperature niches [19]. The importance of tRNA synthetase genes and temperature in the gene analysis suggests these functional differences are linked to energetic requirements [52] (Figs. 2 and 4). Previous work has linked phylogenetic marker genes to environmental processes that may have overestimated the total effect of nutrient conditions on *Prochlorococcus* genomic diversity [15]. This highlights the importance of evaluating both genomic changes along with phylogenetic changes because functional traits may not follow phylogeny due to processes such as horizontal gene transfer and loss of traits through genomic streamlining [53].

Our analysis supports the existence of a novel HLII P-stress haplotype with implications for the mechanism and evolutionary history of microdiversity within *Prochlorococcus*. The organization of phosphorus uptake genes does not follow phylogeny at the broader ecotype level in *Prochlorococcus* and other marine lineages such as *Roseobacter* [25, 54]. This trait organization is common across different microbes and supports the hypothesis that traits organize phylogenetically based on a hierarchy of biochemical complexity, with a “simple” trait like P acquisition being conserved only at the microdiverse level [8, 15, 55]. Our analysis reveals a clear organization of P acquisition genes within the HLII ecotype highlighting the importance of phylogenetic assessment across phylogenetic depths (Figs. 3 and 5 and S3). This phylogenetic organization contrasts with nitrogen acquisition genes, which have not shown any clear within-ecotype organization (Fig. S4) [24, 26]. For nitrogen acquisition genes, it has been hypothesized that vertical inheritance and selection create the systematic distribution of the genes within all *Prochlorococcus*. However, the sporadic distribution within clades may be caused by gene loss and horizontal gene transfer [24]. While P acquisition genes have been found in *Prochlorococcus* phage genomes [28], our analysis suggests the full set of P acquisition genes is phylogenetically conserved in locations of extreme P limitation. Gene abundance of N acquisition genes is variable and changes along a continuous gradient globally, while the presence of P acquisition genes are spatially confined to a few distinct regions [27]. The difference in distributions could suggest different functional and eco-evolutionary processes are acting on P acquisition vs. N acquisition genes. Novel microdiverse sub-taxa can evolve by either the acquisition of a new trait or shifting growth optima along a single trait axis [12]. This might also explain why only 14% of genomic changes could be explained by phylogeny. The rest of the variation may be due to regional differences in N limitation, while P limited regions appear to be stable. These results support our primary nutrient limitation hypothesis, which predicted microdiversity will be driven by differences in adaptation to the primary limiting nutrient and the resulting haplotypes will be globally dispersed (Fig. 1A).

When comparing the amount of variance explained by spatial autocorrelation, we observed a stronger distance decay relationship between gene content than phylogenetic differences (Fig. 4B, C). Microdiverse differences in phylogeny may not show spatial-auto correlation because dispersal overcomes drift in a manner similar to the Bass-Becking hypothesis, “everything is everywhere but the environment selects” [56, 57]. The pattern we observe is indicative of strong contemporary effects combining selection and rapid dispersal resulting in globally conserved microdiverse haplotypes [58]. Alternatively, our metagenomically derived phylogeny may not have the resolution to capture drift between these populations resulting in spatial autocorrelation masked by noisy data.

While metagenomics does allow for many comprehensive analyses, there are also various caveats related to our analyses. To overcome sequencing error and short read length, we used a mapping-based consensus method. This method cannot capture underlying diversity in non-dominant sequence types [59]. *Prochlorococcus* has been shown to have multiple co-existing microdiverse haplotypes in situ, which vary in abundance [43]. Despite these caveats, our parallel analysis of amplicon data in the Indian Ocean suggests the HLII-P haplotype is a single haplotype and the phylogenetic signal is not due to multiple co-existing haplotypes (Fig. S3). There are also some caveats linked to interpreting MAGs. *Prochlorococcus* populations are often made up of multiple closely related haplotypes making it difficult to create complete assemblies that find a consistent path through the assembly graph [60]. While it is most likely that the consistent assembly structure of P acquisition genes in our MAGs is a sign of selection, it could also be a result of divergence in unassembled regions that are not captured in our assemblies. The analysis of new genomes of isolates from other low P environments will help evaluate this caveat.

Connecting microdiversity with specific functional groups has been limited in the field of microbial ecology due to the reliance on amplicon sequencing [61]. Here, we hypothesized that differences in gene content would be primarily driven by nutrient conditions and proposed four hypotheses on the selective pressures that drive *Prochlorococcus* microdiversity. While differences in genome content were better explained by regional differences such as temperature, phylogenetic microdiversity was clearly linked to P limitation conditions with globally conserved haplotypes. This work is an example of how we can leverage large metagenomic datasets to capture global patterns that may otherwise be obscured in a local study. This analysis is the first direct link between phylogenetic microdiversity and functional diversity revealing a novel phylotype linked to nutrient stress. Quantifying the connection between microdiverse haplotypes and traits is important to link microbial processes with larger ecosystem ecology [62].

## Supplementary information

Supplemental figures and tables

Dataset 1

## Data Availability

All sequence data analyzed during the study are publicly accessible through NCBI. Raw metagenomic reads can be found through NCBI: Bio-GO-SHIP BioProject ID PRJNA656268, GEOTRACES BioProject PRJNA385854 and PRJNA385855. Quality controlled MAGs generated in this manuscript are available at 10.5281/zenodo.7950532.

## Code availability

Code used in this manuscript can be found at 10.5281/zenodo.7950482 along with a brief description of usage.
