# Supplementary material for: Global scale phylogeography of functional traits and microdiversity in Prochlorococcus
Source: ISME J. 2023 Jul 15;17(10):1671–9. doi: 10.1038/s41396-023-01469-y (PMC10504305; doi:10.1038/s41396-023-01469-y)
Supplement: Supplementary file 1 — Supplemental figures and tables [file 41396_2023_1469_MOESM1_ESM.docx]

**Supplemental Figures and Tables:**


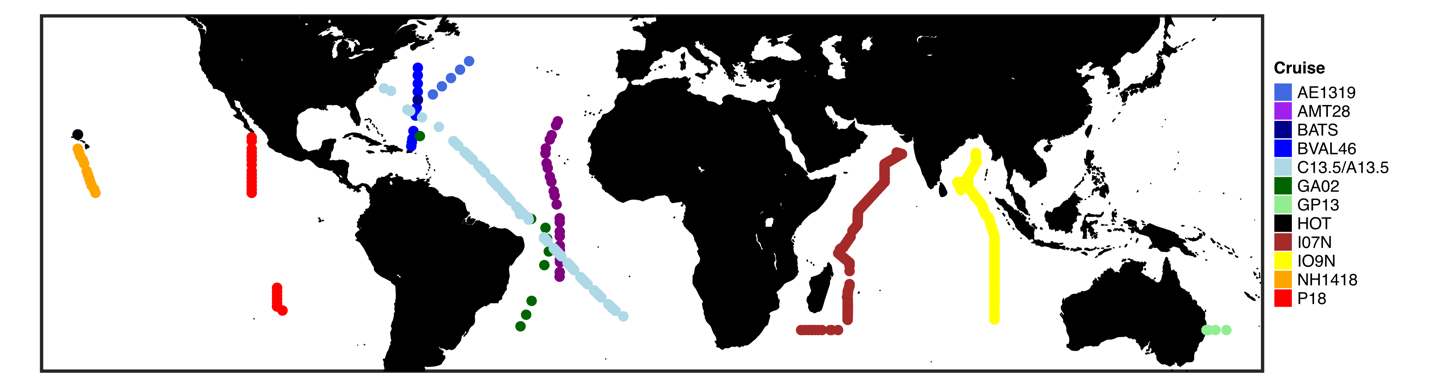


**Figure S1: Map of metagenomic samples colored by cruise track.** Bio-GO-SHIP (AE1319, AMT28, BVAL46, C13.5, IO7N, IO9N, NH1418, P18). GEOTRACES (GA02, GP13).

**Figure S2: Global Variation in Gene Content (Genomic Diversity) of *Prochlorococcus* HLII.** Spatial distribution of principal components based on gene abundance (Figure 2).

**Figure S3: Comparison between amplicon and metagenomic phylogenetic diversity.** **A** Metagenome derived clusters compared to amplicon-based haplotype abundances (Larkin et al. 2020, https://doi.org/10.1002/lno.11251). **B** Boxplot of IO HLII.2 amplicon sequence abundance grouped by metagenomic clusters showing the significant difference between the groups (t-test, *p* = 1.547e-06). **C** Metagenome derived genomic PCA values. **D** Linear comparison between IO HLII.2 relative abundance a metagenome derived genomic PC4. Pearson correlation *r* value and p score are both show in the figure. Color represents the I09 sample number starting with sample 1 at the southernmost part of the transect and the final sample at the northernmost part of the transect (Fig S1).

**Figure S4: Cultured *Prochlorococcus* genomes N and Fe gene annotations.** **A** Clustering of genomes based on N acquisition genes presence and absence. **B** Clustering of genomes based on Fe acquisition genes presence and absence. **C** Phylogenetic tree based on the *rpoC*1 gene with the *Synechococcus* genome GEYO as an outlier. Bootstraps shown at the corresponding node. Gene products can be found in (Table S2).

**Supplemental Tables:**

Table S1: Spearman correlation between genomic PCA analysis, nutrient gene abundances, and environmental factors.

|  | PC1 | PC2 | PC3 | PC4 |
| --- | --- | --- | --- | --- |
| P genes | *p* = 2.2e-16  rho= 0.3877267 | *p* = 0.0001964  rho= 0.147982 | *p* = 0.04191  rho= -0.0810904 | *p* = 2.2e-16  rho= 0.8771648 |
| Fe genes | *p* = 7.31e-06  rho= -0.1775951 | *p* = 5.944e-09  rho= -0.2291705 | *p* = 0.0003255  rho= -0.1427433 | *p* = 2.2e-16  rho= -0.7884059 |
| N genes | *p* = 5.638e-06  rho= -0.1800382 | *p* = 9.578e-08  rho= -0.2110631 | *p* = 2.2e-16  rho= 0.3912358 | *p* = 2.2e-16  rho= -0.4713667 |
| Sea Surface Temp | *p* = 2.2e-16  rho= 0.739164 | *p* = 0.0009222  rho= -0.134813 | *p* = 2.2e-16  rho= -0.3827844 | *p* = 2.2e-16  rho= 0.4637373 |

Table S2: Gene Content PCA (Genomic Diversity) Loadings of Nutrient Uptake and Metabolism Genes of *Prochlorococcus* HLII. PC rank is ordered based on the magnitude of the loading (absolute value).

| Gene | Function | Type | PC1 Rank | PC1 Loading | PC2 Rank | PC2 Loading | PC3 Rank | PC3 Loading | PC4 Rank | PC4 Loading |
| --- | --- | --- | --- | --- | --- | --- | --- | --- | --- | --- |
| *cirA* | Outer membrane receptor for ferrienterochelin and colicins | Fe | 1678 | 0.0106 | 2674 | 0.0017 | 2592 | -0.0021 | 55 | -0.0631 |
| *expD* | Biopolymer transport protein ExbD | Fe | 1617 | 0.0111 | 2659 | -0.0018 | 2579 | 0.0022 | 61 | -0.0613 |
| *febB* | ABC-type Fe3+-hydroxamate transport system, periplasmic component | Fe | 2162 | 0.0070 | 2542 | -0.0028 | 2397 | -0.0036 | 45 | -0.0646 |
| *fepB* | ABC-type cobalamin/Fe3+-siderophores transport system, ATPase component | Fe | 2303 | 0.0061 | 2319 | -0.0047 | 2429 | -0.0033 | 46 | -0.0645 |
| *fepC* | ABC-type Fe3+-siderophore transport system, permease component | Fe | 2246 | 0.0064 | 2259 | 0.0052 | 2046 | -0.0060 | 24 | -0.0676 |
| *fur* | Fe2+ or Zn2+ uptake regulation protein | Fe | 532 | 0.0273 | 127 | 0.0364 | 2172 | -0.0052 | 1531 | -0.0065 |
| *futB* | ABC-type Fe3+ transport system, permease component | Fe | 284 | 0.0320 | 63 | 0.0422 | 2828 | 0.0005 | 2562 | -0.0014 |
| *isiA* | Iron stress-induced chlorophyll-binding protein | Fe | 306 | 0.0315 | 819 | 0.0204 | 61 | -0.0426 | 1181 | -0.0087 |
| *isiB* | Flavodoxin | Fe | 351 | 0.0306 | 2837 | -0.0004 | 1908 | -0.0069 | 323 | -0.0287 |
| *tolQ* | Biopolymer transport protein ExbB/TolQ | Fe | 2231 | 0.0065 | 2541 | -0.0028 | 2742 | 0.0011 | 25 | -0.0672 |
| *tonB* | Lipoprotein-anchoring transpeptidase ErfK/SrfK | Fe | 2365 | 0.0056 | 2385 | -0.0042 | 2026 | -0.0061 | 68 | -0.0591 |
| *amt1* | Ammonia transporter | N | 595 | 0.0260 | 322 | 0.0298 | 199 | -0.0346 | 2134 | -0.0033 |
| *carA* | Carbamoylphosphate synthase small subunit | N | 174 | 0.0345 | 493 | 0.0260 | 1335 | 0.0124 | 2569 | -0.0013 |
| *cynA* | Cyanate transporter | N | 1435 | -0.0124 | 1597 | 0.0116 | 46 | 0.0447 | 264 | -0.0320 |
| *cynS* | Cyanate lyase | N | 1409 | -0.0126 | 1527 | 0.0123 | 40 | 0.0454 | 284 | -0.0311 |
| *dadA* | Glycine/D-amino acid oxidase (deaminating) | N | 477 | 0.0282 | 1840 | -0.0092 | 1189 | 0.0143 | 1689 | -0.0055 |
| *glnA* | Glutamine synthetase | N | 764 | 0.0224 | 31 | 0.0449 | 1146 | -0.0151 | 2423 | 0.0020 |
| *glnB* | Nitrogen regulatory protein PII | N | 262 | 0.0325 | 1065 | -0.0175 | 1583 | 0.0099 | 1411 | -0.0073 |
| *moaA* | Molybdopterin cofactor biosynthesis protein | N | 2744 | 0.0020 | 2768 | -0.0009 | 900 | 0.0189 | 331 | -0.0284 |
| *moaB* | Molybdopterin cofactor biosynthesis protein | N | 2727 | -0.0021 | 2181 | 0.0059 | 838 | 0.0199 | 353 | -0.0266 |
| *moaC* | Molybdopterin cofactor biosynthesis protein | N | 2813 | -0.0011 | 2008 | -0.0077 | 444 | 0.0279 | 274 | -0.0317 |
| *moaE* | Molybdopterin cofactor biosynthesis protein | N | 2575 | 0.0037 | 1290 | -0.0149 | 395 | 0.0294 | 341 | -0.0279 |
| *napA* | Nitrate/nitrite transporter | N | 2886 | 0.0000 | 2581 | -0.0025 | 861 | 0.0195 | 285 | -0.0310 |
| *narB* | Nitrate reductase | N | 2715 | 0.0022 | 2162 | -0.0062 | 692 | 0.0226 | 312 | -0.0290 |
| *nirA* | Nitrite reductase | N | 2851 | -0.0005 | 2811 | -0.0006 | 630 | 0.0238 | 304 | -0.0294 |
| *ntcA* | Nitrogen stress regulator | N | 909 | 0.0194 | 9 | 0.0494 | 1028 | -0.0168 | 2329 | 0.0025 |
| *pipX* | Nitrogen stress regulator | N | 253 | 0.0328 | 2564 | 0.0026 | 1021 | 0.0170 | 2084 | 0.0035 |
| *speA* | Arginine decarboxylase (spermidine biosynthesis) | N | 605 | 0.0258 | 2015 | 0.0077 | 894 | 0.0190 | 1023 | -0.0102 |
| *speB* | Arginase family enzyme | N | 2634 | -0.0031 | 1852 | 0.0091 | 387 | 0.0297 | 217 | -0.0363 |
| *ureA* | Urease gamma subunit | N | 243 | 0.0331 | 917 | -0.0192 | 1076 | 0.0161 | 2186 | -0.0031 |
| *ureB* | Urease beta subunit | N | 252 | 0.0328 | 1314 | 0.0145 | 2624 | -0.0019 | 1722 | -0.0053 |
| *ureC* | Urease alpha subunit | N | 589 | 0.0261 | 95 | 0.0383 | 708 | -0.0224 | 2798 | 0.0004 |
| *ureD* | Urease accessory protein UreH | N | 395 | 0.0297 | 641 | -0.0230 | 184 | 0.0350 | 1409 | -0.0073 |
| *ureE* | Urease accessory protein UreE | N | 547 | 0.0270 | 393 | -0.0280 | 280 | 0.0324 | 975 | -0.0107 |
| *ureF* | Urease accessory protein UreF | N | 398 | 0.0297 | 742 | -0.0216 | 324 | 0.0311 | 2157 | -0.0032 |
| *ureG* | Ni2+-binding GTPase involved in regulation of expression and maturation of urease and hydrogenase | N | 237 | 0.0332 | 871 | 0.0197 | 1644 | 0.0093 | 1287 | -0.0080 |
| *urtA* | ABC-type branched-chain amino acid transport system, periplasmic component | N | 292 | 0.0319 | 586 | 0.0241 | 583 | -0.0248 | 2763 | 0.0006 |
| *acr3* | Arsenite efflux pump ArsB, ACR3 family | P | 2565 | 0.0038 | 1891 | 0.0088 | 319 | 0.0312 | 51 | 0.0635 |
| *arsR* | Putative arsenate stress transcriptional regulator | P | 1135 | 0.0156 | 2009 | 0.0077 | 2262 | -0.0045 | 48 | 0.0643 |
| *chrA* | Transporter, ChrA family | P | 1727 | 0.0103 | 1933 | 0.0083 | 1285 | 0.0131 | 29 | 0.0669 |
| *gap1* | Glyceraldehyde-3-phosphate dehydrogenase | P | 1266 | 0.0140 | 1654 | 0.0109 | 1811 | -0.0078 | 42 | 0.0653 |
| *mfs* | Transporter, multifacilitator family | P | 1400 | 0.0127 | 1525 | 0.0123 | 2123 | -0.0054 | 36 | 0.0663 |
| *phoA* | Alkaline phosphatase (phoA) | P | 2393 | 0.0054 | 1528 | 0.0123 | 734 | 0.0218 | 31 | 0.0667 |
| *phoB* | P stress two-component response regulator | P | 1596 | 0.0113 | 2062 | 0.0072 | 1608 | 0.0096 | 28 | 0.0670 |
| *phoE* | Phosphate uptake outer membrane porin | P | 1193 | 0.0148 | 2135 | 0.0065 | 1903 | -0.0069 | 54 | 0.0632 |
| *phoR* | P stress two-component histidine kinase | P | 1545 | 0.0115 | 2268 | 0.0051 | 1383 | 0.0120 | 27 | 0.0670 |
| *phoX* | Alkaline phosphatase (phoX) | P | 1240 | 0.0142 | 2720 | -0.0014 | 2071 | -0.0058 | 90 | 0.0546 |
| *PMM707* | Hypothetical (upregulated under P stress) | P | 2426 | 0.0052 | 1625 | 0.0112 | 592 | 0.0246 | 37 | 0.0661 |
| *PMM719* | Hypothetical (upregulated under P stress) | P | 1462 | 0.0122 | 1506 | 0.0125 | 188 | 0.0348 | 351 | 0.0273 |
| *PMM721* | Hypothetical (upregulated under P stress) | P | 2561 | 0.0039 | 1636 | 0.0111 | 47 | 0.0446 | 95 | 0.0533 |
| *PMM722* | Hypothetical (upregulated under P stress) | P | 1078 | 0.0163 | 2754 | -0.0011 | 42 | 0.0451 | 178 | 0.0406 |
| *ppA* | Inorganic pyrophosphatase | P | 635 | 0.0252 | 30 | 0.0456 | 830 | -0.0201 | 2173 | -0.0031 |
| *pstS* | ABC-type phosphate transport system, periplasmic component | P | 1129 | 0.0156 | 727 | 0.0218 | 1239 | -0.0136 | 171 | 0.0421 |
| *ptrA* | P stress regulator | P | 2711 | 0.0023 | 1770 | 0.0098 | 189 | 0.0348 | 72 | 0.0582 |
| *unkP1* | Hypothetical (upregulated under P stress) | P | 1841 | 0.0095 | 1551 | 0.0121 | 1677 | 0.0089 | 39 | 0.0656 |
| *unkP2* | Hypothetical (upregulated under P stress) | P | 873 | 0.0202 | 1344 | 0.0141 | 399 | 0.0293 | 107 | 0.0520 |
| *unkP3* | Hypothetical (upregulated under P stress) | P | 2686 | 0.0026 | 1687 | 0.0106 | 351 | 0.0307 | 62 | 0.0610 |
| *unkP4* | Hypothetical (upregulated under P stress) | P | 2625 | -0.0032 | 1657 | 0.0108 | 91 | 0.0399 | 112 | 0.0516 |
| *unkP5* | Hypothetical (upregulated under P stress) | P | 1845 | 0.0094 | 2219 | 0.0055 | 678 | 0.0228 | 18 | 0.0692 |

Table S3: Reference Genome Database

| **Genome** | **Accession** | **Species** |
| --- | --- | --- |
| **EQPAC1** | **GCA_000759875.1** | *Prochlorococcus* |
| **GP2** | **GCA_000759885.1** | *Prochlorococcus* |
| **HNLC1** | **GCA_000218705.1** | *Prochlorococcus* |
| **HNLC2** | **GCA_000218745.1** | *Prochlorococcus* |
| **MED4** | **GCA_000011465.1** | *Prochlorococcus* |
| **MIT0601** | **GCA_000760175.1** | *Prochlorococcus* |
| **MIT0602** | **GCA_000760195.1** | *Prochlorococcus* |
| **MIT0604** | **GCA_000757845.1** | *Prochlorococcus* |
| **MIT0701** | **GCA_000760295.1** | *Prochlorococcus* |
| **MIT0801** | **GCA_000757865.1** | *Prochlorococcus* |
| **MIT1312** | **GCA_001632005.1** | *Prochlorococcus* |
| **MIT1313** | **GCA_001632065.1** | *Prochlorococcus* |
| **MIT1318** | **GCA_001632045.1** | *Prochlorococcus* |
| **MIT1327** | **GCA_001632125.1** | *Prochlorococcus* |
| **MIT1342** | **GCA_001632145.1** | *Prochlorococcus* |
| **MIT9123** | **GCA_000759935.1** | *Prochlorococcus* |
| **MIT9201** | **GCA_000759955.1** | *Prochlorococcus* |
| **MIT9211** | **GCA_000018585.1** | *Prochlorococcus* |
| **MIT9215** | **GCA_000018065.1** | *Prochlorococcus* |
| **MIT9301** | **GCA_000015965.1** | *Prochlorococcus* |
| **MIT9302** | **GCA_000759975.1** | *Prochlorococcus* |
| **MIT9303** | **GCA_000015705.1** | *Prochlorococcus* |
| **MIT9312** | **GCA_000012645.1** | *Prochlorococcus* |
| **MIT9313** | **GCA_000011485.1** | *Prochlorococcus* |
| **MIT9314** | **GCA_000760035.1** | *Prochlorococcus* |
| **MIT9322** | **GCA_000760075.1** | *Prochlorococcus* |
| **MIT9401** | **GCA_000760095.1** | *Prochlorococcus* |
| **MIT9515** | **GCA_000015665.1** | *Prochlorococcus* |
| **NATL1A** | **GCA_000015685.1** | *Prochlorococcus* |
| **NATL2A** | **GCA_000012465.1** | *Prochlorococcus* |
| **PAC1** | **GCA_000760235.1** | *Prochlorococcus* |
| **RS50** | **GCA_001989415.1** | *Prochlorococcus* |
| **SB** | **GCA_000760115.1** | *Prochlorococcus* |
| **SCGCAAA795_I06** | **NA** | *Prochlorococcus* |
| **SCGCAAA795_I15** | **NA** | *Prochlorococcus* |
| **SCGCAAA795_M23** | **NA** | *Prochlorococcus* |
| **SS120** | **GCA_000007925.1** | *Prochlorococcus* |
| **UH18301** | **SAMN00011132** | *Prochlorococcus* |
| **XMU1401** | **GCA_002812945.1** | *Prochlorococcus* |
| **XMU1403** | **GCA_003208065.1** | *Prochlorococcus* |
| **XMU1408** | **GCA_003208055.1** | *Prochlorococcus* |
| **A9spades** | **NA** | *Pelagibacter* |
| **AAA240_E13** | **SAMN02597172** | *Pelagibacter* |
| **AAA288_E13** | **SAMN02597171** | *Pelagibacter* |
| **AAA288_G21** | **SAMN02597281** | *Pelagibacter* |
| **AAA288_N07** | **SAMN02597280** | *Pelagibacter* |
| **AAA298_D23** | **GCA_000402655.1** | *Pelagibacter* |
| **AG_337_G04** | **ERS3879006** | *Pelagibacter* |
| **AG_337_G06** | **ERS3879008** | *Pelagibacter* |
| **AG_426_I15** | **ERS3880727** | *Pelagibacter* |
| **AG_430_F16** | **ERS3880946** | *Pelagibacter* |
| **AG_430_I06** | **ERS3880974** | *Pelagibacter* |
| **B4spades** | **NA** | *Pelagibacter* |
| **F4spades** | **NA** | *Pelagibacter* |
| **HIMB058** | **SAMN02440920** | *Pelagibacter* |
| **HIMB083** | **SAMN02597166** | *Pelagibacter* |
| **HIMB114** | **SAMN02436217** | *Pelagibacter* |
| **HIMB122** | **SRS843558** | *Pelagibacter* |
| **HIMB1321** | **GCA_900177485.1** | *Pelagibacter* |
| **HIMB140** | **ERS787856** | *Pelagibacter* |
| **HIMB4** | **NA** | *Pelagibacter* |
| **HIMB5** | **SAMN00016662** | *Pelagibacter* |
| **HIMB59** | **SAMN00010387** | *Pelagibacter* |
| **HTCC1002** | **SAMN02436088** | *Pelagibacter* |
| **HTCC1013** | **SAMN02441456** | *Pelagibacter* |
| **HTCC1016** | **SAMN02256429** | *Pelagibacter* |
| **HTCC1040** | **SAMN02256395** | *Pelagibacter* |
| **HTCC1062** | **SAMN02603690** | *Pelagibacter* |
| **HTCC7211** | **SAMN02436224** | *Pelagibacter* |
| **HTCC7214** | **SAMN02841172** | *Pelagibacter* |
| **HTCC7217** | **SAMN02841150** | *Pelagibacter* |
| **HTCC8051** | **SAMN02440710** | *Pelagibacter* |
| **HTCC9022** | **SAMN02440781** | *Pelagibacter* |
| **HTCC9565** | **GCA_012932795.1** | *Pelagibacter* |
| **IMCC1322** | **GCA_000024465.1** | *Pelagibacter* |
| **IMCC9063** | **SAMN02603337** | *Pelagibacter* |
| **PRT004** | **NA** | *Pelagibacter* |
| **SAR86B** | **GCA_000252545.1** | *Pelagibacter* |
| **BL107** | **GCF_000153805** | *Synechococcus* |
| **CB0101** | **GCA_000179235.2** | *Synechococcus* |
| **CB0205** | **GCA_000179255.1** | *Synechococcus* |
| **CC9311** | **GCF_000014585** | *Synechococcus* |
| **CC9605** | **GCF_000012625** | *Synechococcus* |
| **CC9616** | **GCF_000515235** | *Synechococcus* |
| **CC9902** | **GCF_000012505** | *Synechococcus* |
| **GEYO** | **GCF_900473955** | *Synechococcus* |
| **GFB01** | **GCA_001039265.1** | *Synechococcus* |
| **KORDI_100** | **GCF_000737535** | *Synechococcus* |
| **KORDI_49** | **GCF_000737575** | *Synechococcus* |
| **KORDI_52** | **GCF_000737595** | *Synechococcus* |
| **MITS9508** | **GCF_001632165** | *Synechococcus* |
| **MITS9509** | **GCF_001631935** | *Synechococcus* |
| **N19** | **GCF_900474045** | *Synechococcus* |
| **N32** | **GCF_900473895** | *Synechococcus* |
| **NKBG042902** | **GCA_000715475.1** | *Synechococcus* |
| **PCC7335** | **GCA_000155595.1** | *Synechococcus* |
| **RCC307** | **GCF_000063525** | *Synechococcus* |
| **REDSEA_S02_B4** | **GCA_001628325.1** | *Synechococcus* |
| **RS9916** | **GCF_000153825** | *Synechococcus* |
| **RS9917** | **GCF_000153065** | *Synechococcus* |
| **UW105** | **GCF_900473935** | *Synechococcus* |
| **UW106** | **GCF_900474015** | *Synechococcus* |
| **UW140** | **GCF_900474295** | *Synechococcus* |
| **UW179A** | **GCF_900473965** | *Synechococcus* |
| **UW179B** | **GCF_900474245** | *Synechococcus* |
| **UW69** | **GCF_900474185** | *Synechococcus* |
| **UW86** | **GCF_900474085** | *Synechococcus* |
| **WH5701** | **GCA_000153045.1** | *Synechococcus* |
| **WH7805** | **GCF_000153285** | *Synechococcus* |
| **WH8016** | **GCF_000230675** | *Synechococcus* |
| **WH8020** | **GCF_001040845** | *Synechococcus* |
| **WH8102** | **GCF_000195975** | *Synechococcus* |
| **WH8109** | **GCF_000161795** | *Synechococcus* |
| **Och114** | **GCA_000014045.1** | *Roseobacter* |

Table S4: Metagenome assembled genome quality control summary table.

| sample | sample consensus *rpoC*1 cluster | percent complete | name | contamination | contains *rpoC*1 |
| --- | --- | --- | --- | --- | --- |
| SRR5720251 | HLII-P | 68% | pro_geo_1 | 1.86% | no |
| SRR5720262 | HLII-P | 61% | pro_geo_2 | 2.99% | no |
| SRR5720332 | HLII-P | 62% | pro_geo_3 | 1.09% | no |
| A392013_303_meta | HLII-P | 63% | pro_AE_1 | 0% | no |
| B462011_061_meta | HLII-P | 49% | pro_BV_1 | 0% | no |
| B462011_152_meta | HLII-P | 60% | pro_BV_2 | 0.54% | no |
| B462011_249_meta | HLII-P | 70% | pro_BV_3 | 3.80% | no |
| B462011_306_meta | HLII-P | 62% | pro_BV_4 | 0.82% | no |
| B462011_474_meta | HLII-P | 60% | pro_BV_5 | 0.28% | no |
| I9N2016_185_meta | HLII-P | 86% | pro_I9_1 | 0.27% | yes |
| I9N2016_187_meta | HLII-P | 78% | pro_I9_2 | 0.27% | no |
| I9N2016_200_meta | HLII-P | 82% | pro_I9_3 | 0.63% | no |
| I9N2016_211_meta | HLII-P | 73% | pro_I9_4 | 0% | no |
| I9N2016_212_meta | HLII-P | 83% | pro_I9_5 | 0.54% | no |
| I9N2016_215_meta | HLII-P | 86% | pro_I9_6 | 1.39% | no |
| I9N2016_218_meta | HLII-P | 52% | pro_I9_7 | 0% | no |
| AMT2018_033_meta | HLII-P | 56% | pro_AM_1 | 0.27% | no |
| AMT2018_051_meta | HLII-P | 47% | pro_AM_2 | 0% | no |
